# Spatio-temporal remodelling of the composition and architecture of the human ovarian cortical extracellular matrix during *in vitro* culture

**DOI:** 10.1093/humrep/dead008

**Published:** 2023-01-31

**Authors:** Johanne Grosbois, Emily C Bailie, Tom W Kelsey, Richard A Anderson, Evelyn E Telfer

**Affiliations:** Institute of Cell Biology, Hugh Robson Building, University of Edinburgh, Edinburgh, UK; Institute of Cell Biology, Hugh Robson Building, University of Edinburgh, Edinburgh, UK; MRC Centre for Reproductive Health, Queens Medical Research Institute, University of Edinburgh, Edinburgh, UK; School of Computer Science, University of St Andrews, St Andrews, UK; MRC Centre for Reproductive Health, Queens Medical Research Institute, University of Edinburgh, Edinburgh, UK; Institute of Cell Biology, Hugh Robson Building, University of Edinburgh, Edinburgh, UK

**Keywords:** fertility preservation, extracellular matrix, ovary, mechanobiology, tissue stiffness, primordial follicle activation

## Abstract

**STUDY QUESTION:**

How does *in vitro* culture alter the human ovarian cortical extracellular matrix (ECM) network structure?

**SUMMARY ANSWER:**

The ECM composition and architecture vary in the different layers of the ovarian cortex and are remodelled during *in vitro* culture.

**WHAT IS KNOWN ALREADY:**

The ovarian ECM is the scaffold within which follicles and stromal cells are organized. Its composition and structural properties constantly evolve to accommodate follicle development and expansion. Tissue preparation for culture of primordial follicles within the native ECM involves mechanical loosening; this induces undefined modifications in the ECM network and alters cell–cell contact, leading to spontaneous follicle activation.

**STUDY DESIGN, SIZE, DURATION:**

Fresh ovarian cortical biopsies were obtained from six women aged 28–38 years (mean ± SD: 32.7 ± 4.1 years) at elective caesarean section. Biopsies were cut into fragments of ∼4 × 1 × 1 mm and cultured for 0, 2, 4, or 6 days (D).

**PARTICIPANTS/MATERIALS, SETTING, METHODS:**

Primordial follicle activation, stromal cell density, and ECM-related protein (collagen, elastin, fibronectin, laminin) positive area in the entire cortex were quantified at each time point using histological and immunohistological analysis. Collagen and elastin content, collagen fibre characteristics, and follicle distribution within the tissue were further quantified within each layer of the human ovarian cortex, namely the outer cortex, the mid-cortex, and the cortex–medulla junction regions.

**MAIN RESULTS AND THE ROLE OF CHANCE:**

Primordial follicle activation occurred concomitantly with a loosening of the ovarian cortex during culture, characterized by an early decrease in stromal cell density from 3.6 ± 0.2 × 10^6^ at day 0 (D0) to 2.8 ± 0.1 × 10^6^ cells/mm^3^ at D2 (*P* = 0.033) and a dynamic remodelling of the ECM. Notably, collagen content gradually fell from 55.5 ± 1.7% positive area at D0 to 42.3 ± 1.1% at D6 (*P* = 0.001), while elastin increased from 1.1 ± 0.2% at D0 to 1.9 ± 0.1% at D6 (*P* = 0.001). Fibronectin and laminin content remained stable. Moreover, collagen and elastin distribution were uneven throughout the cortex and during culture. Analysis at the sub-region level showed that collagen deposition was maximal in the outer cortex and the lowest in the mid-cortex (69.4 ± 1.2% versus 53.8 ± 0.8% positive area, respectively, *P* < 0.0001), and cortical collagen staining overall decreased from D0 to D2 (65.2 ± 2.4% versus 60.6 ± 1.8%, *P* = 0.033) then stabilized. Elastin showed the converse distribution, being most concentrated at the cortex–medulla junction (3.7 ± 0.6% versus 0.9 ± 0.2% in the outer cortex, *P* < 0.0001), and cortical elastin peaked at D6 compared to D0 (3.1 ± 0.5% versus 1.3 ± 0.2%, *P* < 0.0001). This was corroborated by a specific signature of the collagen fibre type across the cortex, indicating a distinct phenotype of the ovarian cortical ECM depending on region and culture period that might be responsible for the spatio-temporal and developmental pattern of follicular distribution observed within the cortex.

**LARGE SCALE DATA:**

N/A.

**LIMITATIONS, REASONS FOR CAUTION:**

Ovarian cortical biopsies were obtained from women undergoing caesarean sections. As such, the data obtained may not accurately reflect the ECM distribution and structure of non-pregnant women.

**WIDER IMPLICATIONS OF THE FINDINGS:**

Clarifying the composition and architecture signature of the human ovarian cortical ECM provides a foundation for further exploration of ovarian microenvironments. It is also critical for understanding the ECM–follicle interactions regulating follicle quiescence and awakening, leading to improvements in both *in vitro* activation and *in vitro* growth techniques.

**STUDY FUNDING/COMPETING INTEREST(S):**

Medical Research Council grant MR/R003246/1 and Wellcome Trust Collaborative Award in Science: 215625/Z/19/Z. The authors have no conflicts to declare.

**TRIAL REGISTRATION NUMBER:**

N/A.

## Introduction

Primordial follicles are the most abundant population of ovarian follicles and represent the functional unit of the mammalian ovary. The majority of them are quiescent at any time, and in women this can be for decades, ensuring a prolonged reproductive lifespan. From primordial follicle formation until menopause, resting follicles are recruited into the growing pool, supplying the ovary with its population of growing follicles. This process of folliculogenesis makes the ovary one of the most dynamic organs of the human body during the reproductive life span of women.

The repetitive processes of follicular development and degeneration (atresia), ovulation, corpus luteum formation, and regression require extensive structural remodelling during each reproductive cycle, provided by its extracellular matrix (ECM) (Ny *et al.*, 2002; Smith *et al.*, 2002). The ovarian ECM is a cell–surface-associated macromolecular network that forms the 3D scaffold in which follicles and stromal cells reside. It is composed of an interlocking mesh of water, proteoglycans, and fibrous proteins secreted by resident cells (Theocharis *et al.*, 2016). Recent proteomic studies of the human ovarian cortex identified up to 120 proteins related to the matrisome, defined as the ensemble of both structural ECM components and ECM-associated proteins known to regulate and remodel the ECM (Ouni *et al.*, 2019, 2022). Among them, collagen subtypes, providing tissues with physical support and mechanical properties such as strength and rigidity (Tang, 2020), were highly represented and constituted 49% of the total matrisome. Glycoproteins, including elastin, regulating tissue elasticity and resilience (Vindin *et al.*, 2019), fibronectin, controlling cell adhesion, migration, proliferation, and differentiation (Zollinger and Smith, 2017), and laminin, a major component of basement membranes and a key component for cell attachment and ECM organization (Aumailley, 2013), represented 15% of the identified proteins (Ouni *et al.*, 2019).

Components of the ECM link together to form a structurally stable composite, providing tissues with physical support for the cellular constituents as well as biochemical and biomechanical properties that are required for tissue morphogenesis, differentiation, and homeostasis (Theocharis *et al.*, 2016). The ECM is also a reservoir of growth factors and bioactive molecules, such as hormones and growth factors, regulating spatially and temporally their diffusion and availability within the ovarian niche (Taipale and Keski-Oja, 1997; Bonnans *et al.*, 2014). Furthermore, growing evidence suggests ECM involvement during folliculogenesis, particularly during primordial follicle activation. First, the ovary is divided into two main compartments, the cortex, containing mostly quiescent follicles, and the medulla, harbouring developing follicles. This compartmentalization is determined by the spatial ECM composition, organization, and density. In large mammals, including humans, it is believed that the stiff collagen-rich cortical region provides a rigid physical environment that maintains quiescence, while the softer medulla layer offers a more pliant environment that enables follicle expansion and growth (Woodruff and Shea, 2011). The various degrees of mechanical forces imposed and interpreted by the follicular cells through mechano-sensing may contribute to regulating the balance between follicular quiescence and activation. Second, it has been demonstrated that mouse oocytes in primordial follicles are compressed by surrounding granulosa cells secreting ECM proteins, leading to a state of high mechanical stress (Nagamatsu *et al.*, 2019). Conversely, loosening of the ovarian ECM using a collagenase‐containing solution triggered follicle activation, while compression with exogenous pressure restored follicle dormancy (Nagamatsu *et al.*, 2019). Third, physically disruptive methods that loosen the tissue, such as ovarian fragmentation or drilling, are increasingly being used in clinical settings to release surface tension and disrupt the Hippo pathway, relieving inhibition of follicle activation and growth in women with premature ovarian insufficiency (POI) and polycystic ovary syndrome (PCOS), respectively (Devos *et al.*, 2022). Fourth, *in vitro* studies of isolated murine and primate follicles grown in alginate hydrogels of varying concentrations further confirm that a stiff environment is necessary to maintain primordial follicle quiescence and survival, but negatively affects secondary follicle growth, steroid production, and meiotic potential (Xu *et al.*, 2006; West *et al.*, 2007; Hornick *et al.*, 2012). These findings imply that a reduced mechanical stress would create a more permissive environment, more favourable for primordial follicle activation and growth of good quality oocytes.

The development of multi-step *in vitro* culture systems that support the activation and growth of early-stage follicles up to maturity has the potential to be a source of mature eggs for IVF (Grosbois *et al.*, 2022). Fertilizable eggs and live pups have already been obtained from cultured primordial follicles in mice (Eppig and O'Brien, 1996; O'Brien *et al.*, 2003), and significant progress has been made in culturing human follicles, as evidenced by mature oocytes being derived from *in vitro* grown primordial/unilaminar follicles (McLaughlin *et al.*, 2018; Xu *et al.*, 2021). Nevertheless, the development of mature oocytes from *in vitro* cultured follicles is inefficient, with a major obstacle to improvement being the substantial knowledge gap regarding the underlying mechanisms controlling follicle activation. Primordial follicles isolated from human ovarian tissue are not activated to grow *in vitro* (Abir *et al.*, 1999), but spontaneous activation is achieved when follicles are maintained within small pieces of ovarian cortex containing stromal cells (Hovatta *et al.*, 1997; Wright *et al.*, 1999; Telfer *et al.*, 2008; Garor *et al.*, 2009). Clearly, preserving the structural integrity of the tissue and the physical interactions between follicles and their surrounding environment is essential for follicle activation, yet the contribution of the ovarian stroma to regulating follicle quiescence and growth is poorly defined. We hypothesized that culture-induced follicular activation and early growth could be a consequence of a dynamic remodelling of the ovarian ECM, potentially leading to the creation of a more permissive microenvironment. In this study, we explored the changes of the ECM composition and architectural features in each region of the ovarian cortex together with follicle dynamics and localization during culture.

## Materials and methods

### Ethical approval

Approval of this study to obtain ovarian cortical biopsies after informed consent from women undergoing elective caesarean section was given by the local ethics committee (ref LREC 10/S1101/2).

### Ovarian tissue collection and preparation

Fresh ovarian cortical biopsies were obtained from six women aged 28–38 years (mean ± SD: 32.7 ± 4.1 years) undergoing elective caesarean section. Ovarian tissue was transported to the laboratory in dissection medium [Leibovitz medium (Invitrogen Ltd, Paisley, UK) supplemented with sodium pyruvate (2 mM), glutamine (2 mM) (both Invitrogen Ltd), human serum albumin (HSA) (3 mg/ml), penicillin G (75 μg/ml), and streptomycin (50 μg/ml) (Sigma Chemicals, Poole, Dorset UK)]. At the laboratory, excess stromal and haemorrhagic tissue as well as follicles measuring >80 μm were removed and the ovarian cortex was mechanically loosened, as previously described (Telfer and McLaughlin, patent EP3198005B1). Briefly, the tissue was anchored to the base of the petri dish with a needle, and using the blunt edge of a scalpel blade, gentle pressure was applied along the tissue surface, stretching the tissue away from the anchor point such that the size of the cortical sample is increased by at least 10%. The cortex was then cut into fragments of ∼4 × 1 mm and ∼1 mm thick.

### Ovarian tissue culture

One to three fragments were selected from each biopsy as Day 0 (D0) controls. The remaining fragments (two to three per day of culture) were incubated individually in 24-well cell culture plates (Corning B.V. Life Sciences Europe, Amsterdam) as previously described (Telfer *et al.*, 2008; McLaughlin *et al.*, 2018). Briefly, 300 μl of culture medium was added per well [McCoy’s 5a medium with bicarbonate supplemented with HEPES (20 mM; Invitrogen Ltd), glutamine (3 mM; Invitrogen Ltd), HSA (0.1%), penicillin G (0.1 mg/ml), streptomycin (0.1 mg/ml), transferrin (2.5 μg/ml), selenium (4 ng/ml), human insulin (10 ng/ml), 1 ng/ml recombinant human FSH, and ascorbic acid (50 μg/ml) (all obtained from Sigma Chemicals, UK, unless specified)]. Fragments were cultured for up to 6 days at 37°C in humidified air with 5% CO_2_ with half the media being removed and replaced every second day. After 0, 2, 4, or 6 days of culture, the ovarian fragments were fixed in 10% normal buffered formalin for histological and immunohistological evaluation.

### Histological analysis

Fixed ovarian fragments were dehydrated in increasing concentrations of ethanol (70–100%), embedded in paraffin and serially sectioned at 5 μm thickness. Every fifth section was stained with haematoxylin and eosin, and both follicle counts and stromal cell density were assessed. Follicles were classified according to their developmental stage as primordial follicles (oocyte surrounded by a few flattened granulosa cells), transitory follicles (oocyte surrounded by flattened and at least one cuboidal granulosa cell), or growing follicles (oocyte surrounded by one or more complete layer(s) of cuboidal granulosa cells). Only follicles that contained an oocyte nucleus were counted to prevent double counting. Follicle spatial distribution within each sub-region of the ovarian cortex, namely the outer cortex, the mid-cortex, and the cortex–medulla junction, was assessed by dividing the cortex into three equal layers of 300 µm each from the epithelium surface to the medulla side. The outer cortex and cortex–medulla junction regions were defined in the sections based on the identification of the surface epithelium and tunica albuginea layers on one side, and small cortical arterioles on the opposite side, respectively.

For PicroSirius Red (PSR) staining, sections were deparaffinized and rehydrated in a series of ethanol baths of decreasing concentrations. The slides were immersed in a PSR staining solution (ab246832, Abcam, UK) for 1 h at room temperature, then washed twice with 0.5% glacial acetic acid and three times with 100% ethanol. The slides were cleared in xylene and mounted with DPX. All slides from the same patient were processed at the same time to minimize staining variation.

### Immunostainings of ECM components

Immunohistochemistry and immunofluorescence were performed for the evaluation of various ECM components. Briefly, after deparaffinization and rehydration, slides to be stained for laminin, fibronectin, and elastin were immersed in Tris-EDTA buffer (pH 8.5) for 40 min at 95°C, then immersed in 3% (v/v) hydrogen peroxide to quench endogenous peroxidase activity. Non-specific binding sites were blocked using 5% normal goat serum for 1 h at room temperature. Sections were subsequently incubated overnight at 4°C with Anti-LAMB2 (Atlas antibodies, cat#HPA001895, 1:100), Anti-FN1 (Atlas antibodies, cat#HPA027066, 1:200), or Anti-elastin (Abcam, ab213720, 1/200). The next day, slides targeting laminin and fibronectin were incubated for 1 h at room temperature with a biotinylated secondary antibody (Vectastain Elite ABC kit; Vector Laboratories, Peterborough, UK), rinsed twice then incubated with Streptavidin Horseradish Peroxidase for 30 min at room temperature. After washing, the signal was visualized using 3,3′-diaminobenzidine, then counterstained with haematoxylin, dehydrated and mounted with DPX. Slides targeting elastin were incubated with an Alexa Fluor 488-conjugated goat anti-rabbit secondary antibody (A11034; Invitrogen) for 1 h at room temperature, washed then mounted in Vectashield with DAPI (Vector Labs). Negative controls were obtained by replacing the primary antibody with blocking solution containing non-immune serum, while human testis was used for positive controls of all tested antibodies (Supplementary Fig. S1). All washes were performed in PBS or PBS with 0.05% (v/v) Tween 20 at room temperature. All slides from the same patient were processed at the same time to minimize staining variation.

### Image analysis

Brightfield images of 15 random fields (315 × 235 × 5 μm = 370 125 μm^3^) per day of culture and per patient were captured using an inverted microscope (Leica DMIRB) equipped with Retiga 2000R camera and QCapturePro software (Teledyne Photometrics UK Ltd, Essex, UK). Stromal cell nuclei were counted using FIJI (Image J) and the mean stromal cell density was calculated by dividing the stromal cell number by the volume of tissue analysed.

Brightfield (PSR, fibronectin, laminin) and fluorescent (elastin) images of entire cortical sections or each layer of the human ovarian cortex, namely the outer cortex, the mid-cortex, and the cortex–medulla junction regions, were captured using a light microscope (Leica) at ×2.5 (PSR, fibronectin, laminin) and ×5 (elastin) or ×40 magnifications (PSR, elastin). Quantification of the average area of positive staining per condition and per patient was performed using FIJI.

PSR samples were further analysed under polarized light for bulk (total) and individual fibre assessment of collagen features. Birefringent images were acquired using an Axiovert 200 microscope (Carl Zeiss, Oberkochen, Germany) at ×40 magnification. Collagen fibre colour, which reflects fibre diameter and packing density, was quantified using a custom FIJI macro. Briefly, the hue (colour) of each pixel was determined and a colour threshold was used to isolate the three main colours seen in PSR-stained samples under polarized light: red (thick fibres), yellow (mid-sized fibres), and green (thin fibres). In order to streamline the data obtained and juxtapose them with previously described collagen measurements in the ovary, the thresholds on the hue histogram were set as follows, based on Ouni *et al.* (2020): red 2–9, yellow/orange 10–38, and green 24–135. The relative percentage of each colour was calculated by dividing the pixel count of each colour by the total pixel count for each image. Collagen fibre metrics were investigated with the curvelet transform-fibre extraction (CT-FIRE) and CurveAlign (v4.0) programs (LOCI; Madison, WI, USA) from 137 × 137 µm^2^ regions of interest across the ovarian cortex. CT-FIRE was applied to overlay each collagen fibre and then extract information of the individual fibre characteristics including their length, width, straightness, and angle, while CurveAlign was used to overlay each collagen fibre, which was then converted into a direction heat map, allowing for quantification of the coefficient of alignment.

Fibre density was calculated as the total number of fibres per 100 µm^2^; the straightness coefficient represents how close the shape of the fibre is to straight line and ranges from 0 to 1, where 1 indicates a perfectly straight fibre and lower values indicate curvier fibres; fibre angle defines the angle between the horizontal axis and a straight line between the fibre’s curve endpoints, ranging between 0° and 180°; and the alignment coefficient indicates the dispersion of the fibre orientations on a scale from 0 to 1, with 1 indicating perfectly aligned fibres, and smaller values representing more randomly distributed fibres. Images were processed as ≈1000 fibres per image; three images per patient per group; six patients per group; and >17 000 fibres analysed per group.

### Statistical analysis

Analysis was performed using SPSS v25 (IBM Corp., Armonk, NY, USA). Normality was verified by the Levene test then one-way ANOVA followed by Tukey’s *post hoc* least significant difference multiple comparisons was applied for the analysis of follicle activation, stromal cell density, and the percentage of ECM components positive area in the overall tissue. The study was based on a limited number of patients, and it is acknowledged that natural biological variation exists in human samples. Thus, for multivariable analyses, we first calculated the random effects variance in primordial follicle density using the intraclass correlation coefficients (ICCs) (Abbara *et al.*, 2021) (Supplementary Fig. S2). ICC was 0.144, indicating that 14.4% of the variation in the percentage of primordial follicle density can be attributed to the random effect of subject difference, with this low value suggesting that standard regression models and multi-level analysis methods will have similar efficacy for these data. Hence multivariable linear regression, univariate and multivariate general linear models were used to determine the effect of both the culture period and the cortical sub-region on ECM composition and its architectural features, and on follicle geographical distribution within the stroma. Data are presented as box plots or mean ± SEM. A value of *P* < 0.05 was considered statistically significant.

## Results

### Follicles spontaneously activate and grow during *in vitro* culture

**Figure 1.**
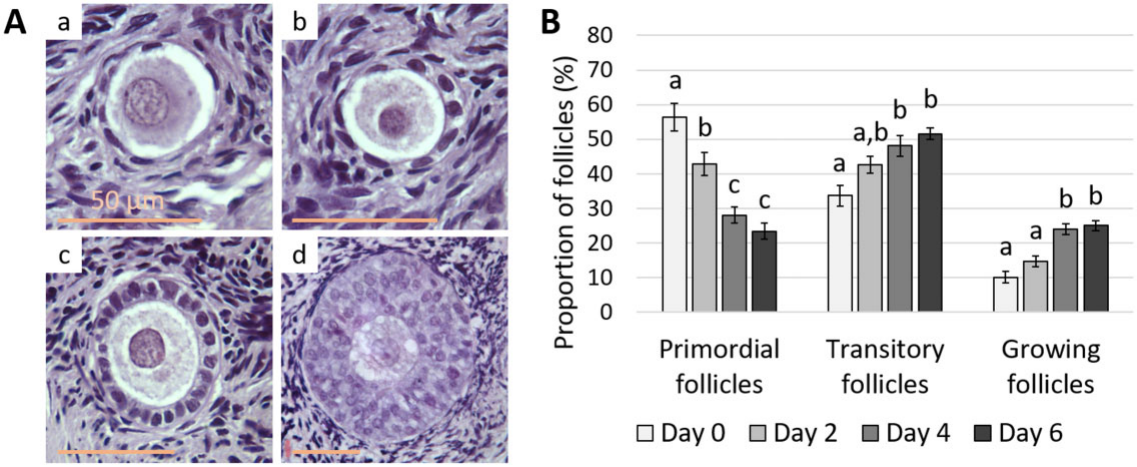
**Primordial follicle activation and early growth during *in vitro* culture.** (**A**) Photomicrographs of human primordial (**a**), transitory (**b**), primary (**c**), and secondary (**d**) follicles after haematoxylin and eosin staining. Scale bar = 50 µm. (**B**) Distribution of the follicles according to their developmental stage at 0, 2, 4, and 6 days of culture (n = 6; 5703 follicles counted). Data are expressed as mean ± SEM, ANOVA followed by Tukey test. Different superscripts indicate *P* < 0.05.

A total of 5703 follicles from six patients were examined under the light microscope and classified according to their developmental stage (Fig. 1A). Primordial follicles were the most prevalent at D0, constituting 56.4 ± 3.9% of the total follicle number, while 33.6 ± 3.1% and 10.0 ± 1.7% of the follicles were at the transitory and growing stage of development, respectively. Quiescent follicles were spontaneously recruited into the transitory and growing pools during culture (Fig. 1B). The proportion of primordial follicles gradually declined from 56.4 ± 3.9% to 23.4 ± 2.3% after 6 days of culture (*P* < 0.0001), and this drop was balanced by a significant increase in the percentage of both transitory follicles from 33.6 ± 3.1% to 51.6 ± 1.6% and growing follicles from 10.0 ± 1.7% to 25.0 ± 1.5% (*P* = 0.001 and *P* = 0.0004, respectively). These results confirmed the culture-induced remodelling at the follicular level, so we deepened our exploration of the ovarian cortical stromal tissue and its ECM.

### Loosening of the ovarian cortex during culture

**Figure 2.**
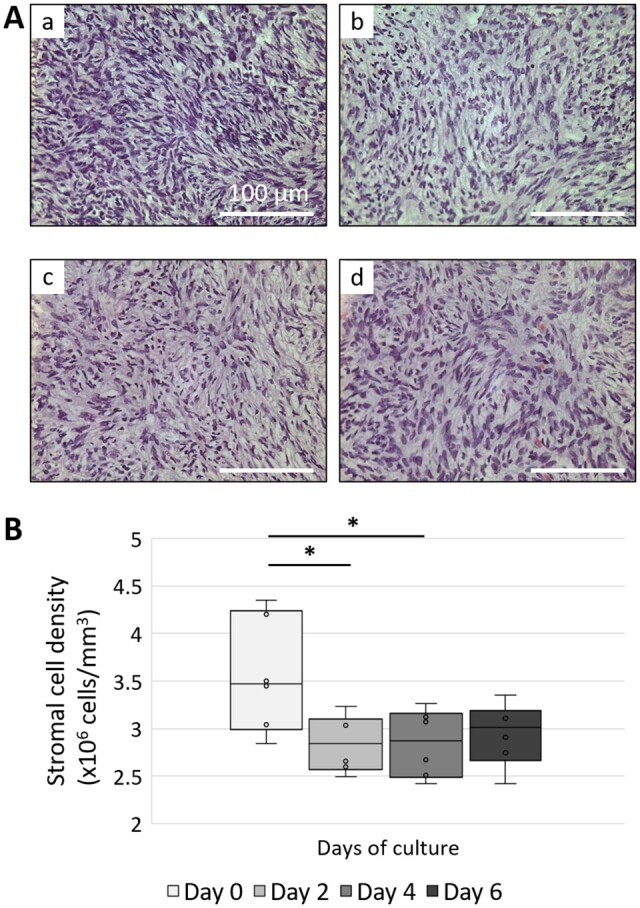
**Distribution of the stromal cells within the human ovarian cortex during culture.** (**A**) Representative images of the ovarian stroma at 0 (**a**), 2 (**b**), 4 (**c**), and 6 (**d**) days of culture after haematoxylin and eosin staining. Scale bar = 100 µm. (**B**) Box plots of stromal cell density (n = 6). ANOVA followed by Tukey test, *P < 0.05.

Stromal cell density is an important indicator of tissue integrity and is likely to play a role in folliculogenesis through cellular mechano-sensing. We measured quantitatively the density of stromal cells within the cortex during culture and found it to decrease from 3.6 ± 0.2 × 10^6^ cells/mm^3^ at D0 to 2.8 ± 0.1 × 10^6^ cells/mm^3^ at D2 (*P* = 0.033) and then remained stable throughout the remainder of the culture period (Fig. 2).

### Temporal remodelling of the ECM components of the ovarian cortex during *in vitro* culture

**Figure 3.**
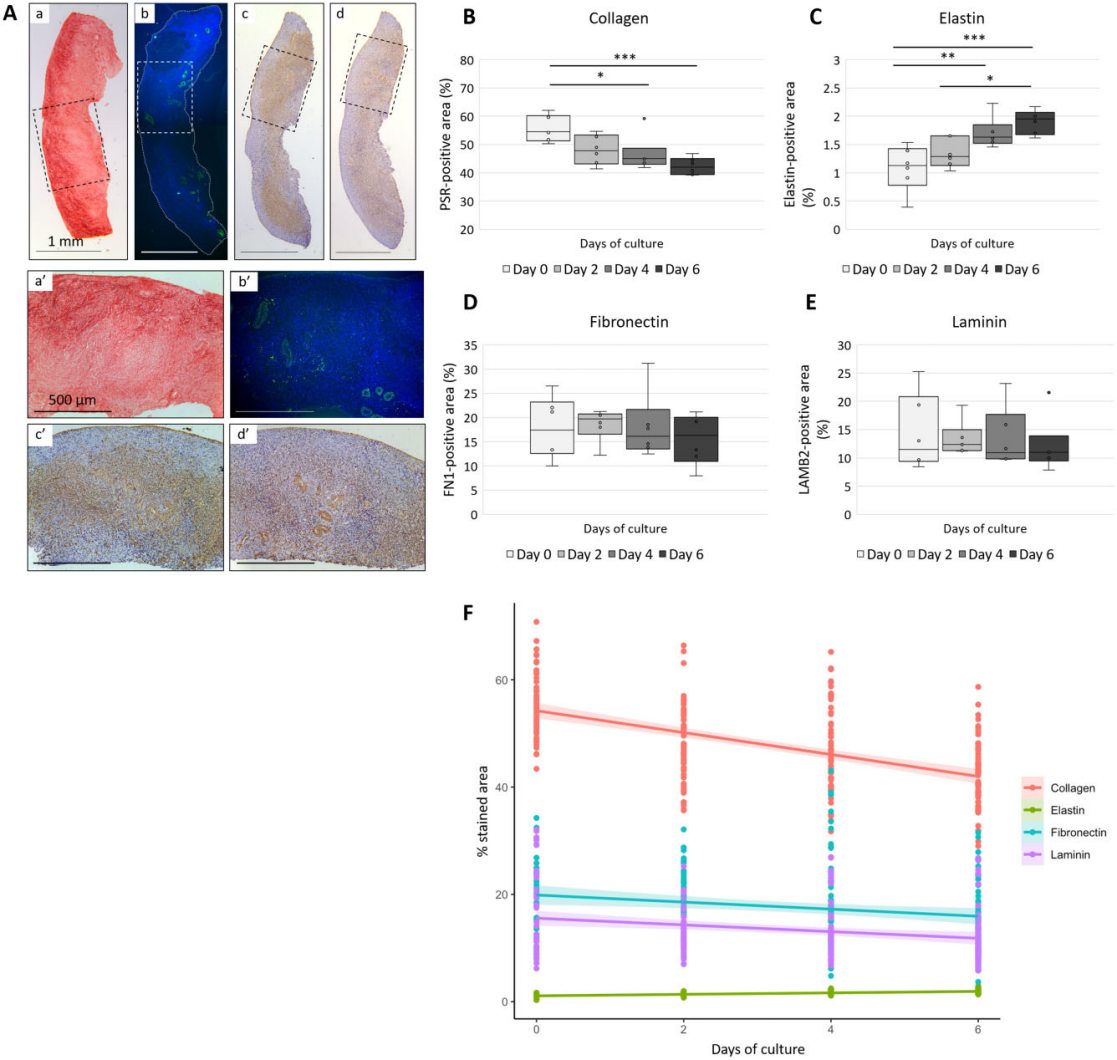
**Dynamics of collagen, elastin, fibronectin, and laminin proteins during *in vitro* culture.** (**A**) Representative images of whole human ovarian cortex (2.5× magnification, **a**–**d**) and at higher magnification (10× magnification, **a’**–**d’**) of PicroSirius Red stained collagen (a, a’) and immunostained elastin (b, b’), fibronectin (c, c’), and laminin (d, d’). Collagen fibres stain in red, elastin in green, and DAPI in blue, fibronectin and laminin in brown. Scale bar = 1 mm (a–d) and 500 µm (a’–d’). (**B**–**E**) Quantification of the area of extracellular matrix (ECM)-related proteins positive staining per day of culture (n = 6). Data presented as box plots, ANOVA followed by Tukey test. *P < 0.05, **P < 0.01, ***P < 0.001. (**F**) Summary of the remodelling of all studied ECM component proteins in the human ovarian cortex during *in vitro* culture. For each protein, lines of best fit with 95% CI as shaded regions are represented.

ECM remodelling during culture was evaluated by tracking the dynamics of four of its components, namely collagen, elastin, fibronectin, and laminin, in entire ovarian cortical sections. Collagen deposition was predominantly visible at the outer edge of the ovary and reduced towards the inner side (Fig. 3A). Collagen protein staining gradually declined during culture, from 55.5 ± 1.7% positive area at day 0 (D0) to 42.3 ± 1.1% at D6 (*P* = 0.001) (Fig. 3B). In contrast, elastin was mostly located at the cortex–medulla border, particularly near blood vessels, and to a lesser extent within the cortical stroma, while it was barely visible in the more superficial parts of the cortex as well as the surface epithelium (Fig. 3A). Elastin protein staining increased from 1.1 ± 0.2% at D0 to 1.9 ± 0.1% at D6 (*P* = 0.001) (Fig. 3C). Fibronectin and laminin were diffusely expressed throughout the stromal compartment (Fig. 3A) and levels remained stable throughout culture (fibronectin: 17.8 ± 2.4% at D0 versus 15.6 ± 2.0% at D6; *P* = 0.90; laminin: 14.3 ± 2.5% at D0 to 12.1 ± 1.8% at D6; *P* = 0.88) (Fig. 3D and E). Taken together, these results clearly illustrate ovarian cortical ECM remodelling during *in vitro* culture (Fig. 3F and Supplementary Fig. S3).

### Distinct collagen and elastin phenotypes across the ovarian cortex during culture

**Figure 4.**
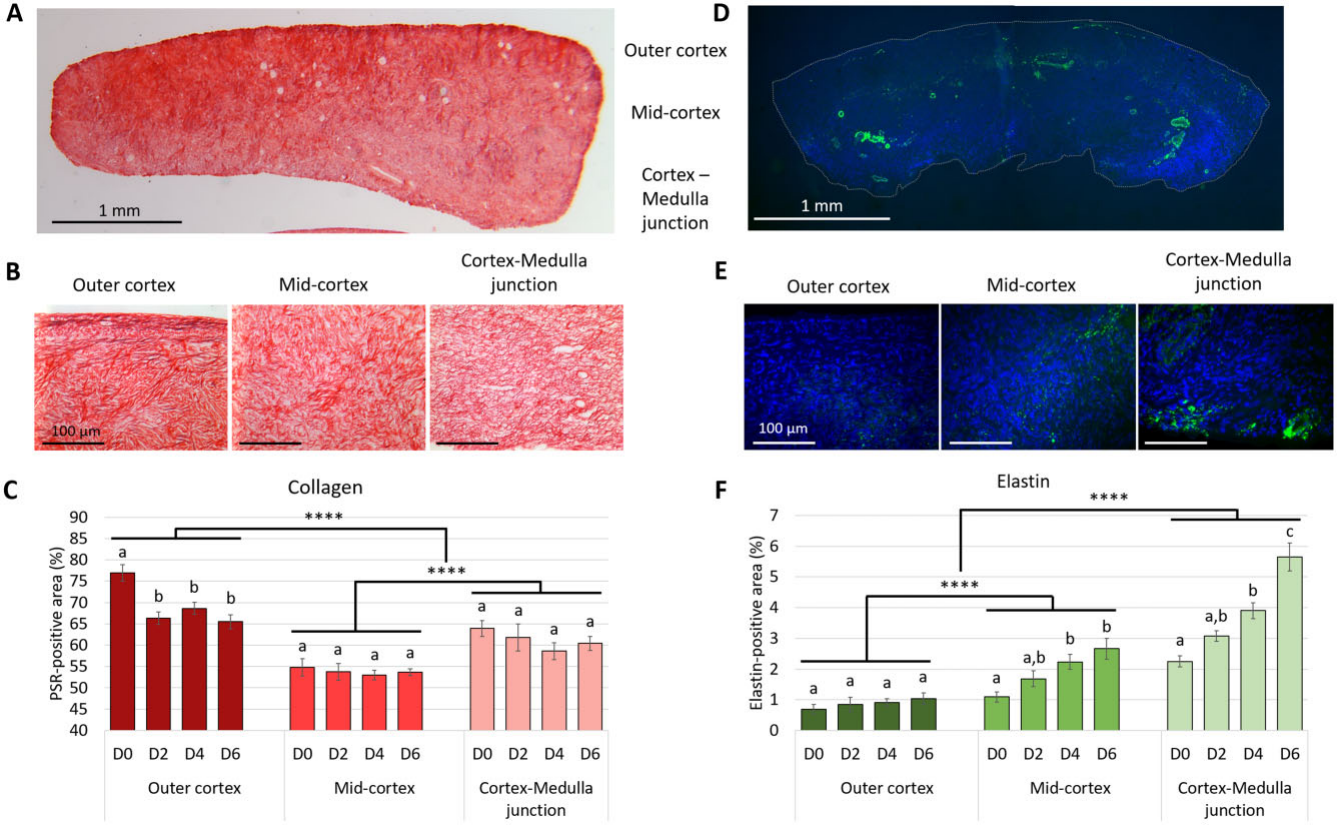
**Cortical sub-regions- and culture-induced collagen and elastin remodelling.** (**A**–**C**) collagen, (**D**–**F**) elastin. (A, D) Representative images of entire human cortical sections stained with PicroSirius Red (A) or elastin (D). Collagen fibres stain in red, elastin in green, and DAPI in blue. Scale bar = 1 mm. (B, E) Selected regions of interest within the outer cortex, the mid-cortex, and the cortex–medulla junction sub-regions. Scale bar = 100 µm. (C, F) Quantification of the area of PicroSirius Red- (C) and elastin- (F) positive staining depending on the cortical sub-region and the culture period (n = 6). Data are expressed as mean ± SEM, univariate general linear models. Different superscripts indicate *P* < 0.05 within each of the cortical sub-region; ****P < 0.0001.

Given the uneven distribution of collagen and elastin observed throughout the ovarian cortex, we further quantified their spatial diffusion at different cortical sub-regions, namely the outer cortex, the mid-cortex, and the cortex–medulla junction, throughout the culture period. Collagen and elastin were differentially distributed both throughout the cortex and during culture (Fig. 4). Collagen deposition was maximal at the outer cortex and the lowest at the mid-cortex (69.4 ± 1.2% versus 53.8 ± 0.8% positive area, respectively *P* < 0.0001), and decreased from D0 to D2 (65.2 ± 2.4% versus 60.6 ± 1.8% positive area, *P* = 0.033) then stabilized. The decline in collagen over time was localized to the outer cortex, dropping from 77.0 ± 1.9% positive staining at D0 to 66.3 ± 1.5% at D2 (*P* = 0.002) then stabilizing, while collagen remained stable in both the mid-cortical and the cortex–medulla junction collagen throughout culture (54.8 ± 2.1% at D0 versus 53.7 ± 0.8% at D6; *P* = 0.965 and 63.9 ± 1.9% at D0 to 60.4 ± 1.6% at D6; *P* = 0.828, respectively) (Fig. 4C). Conversely, elastin content was concentrated at the cortex–medulla junction and gradually decreased towards the ovarian surface (3.7 ± 0.6% versus 0.9 ± 0.2% positive area, respectively *P* < 0.0001), and peaked at D6 compared to D0 (3.1 ± 0.5% versus 1.3 ± 0.2% positive area, *P* < 0.0001). The elastin increase was particularly visible at the medullary border, rising from 2.2 ± 0.2% positive staining at D0 to 5.6 ± 0.5% at D6 (*P* < 0.0001), and more moderately at the mid-cortex, from 1.1 ± 0.2% to 2.7 ± 0.3% (*P* = 0.004), while its level remained stable at the outer cortex (0.7 ± 0.2% versus 1.0 ± 0.2%, *P* = 0.64) (Fig. 4F). These data indicate a distinct phenotype of the ovarian cortical ECM related to both region and culture period.

### Specific signature of the collagen fibres metrics across the ovarian cortex

**Figure 5.**
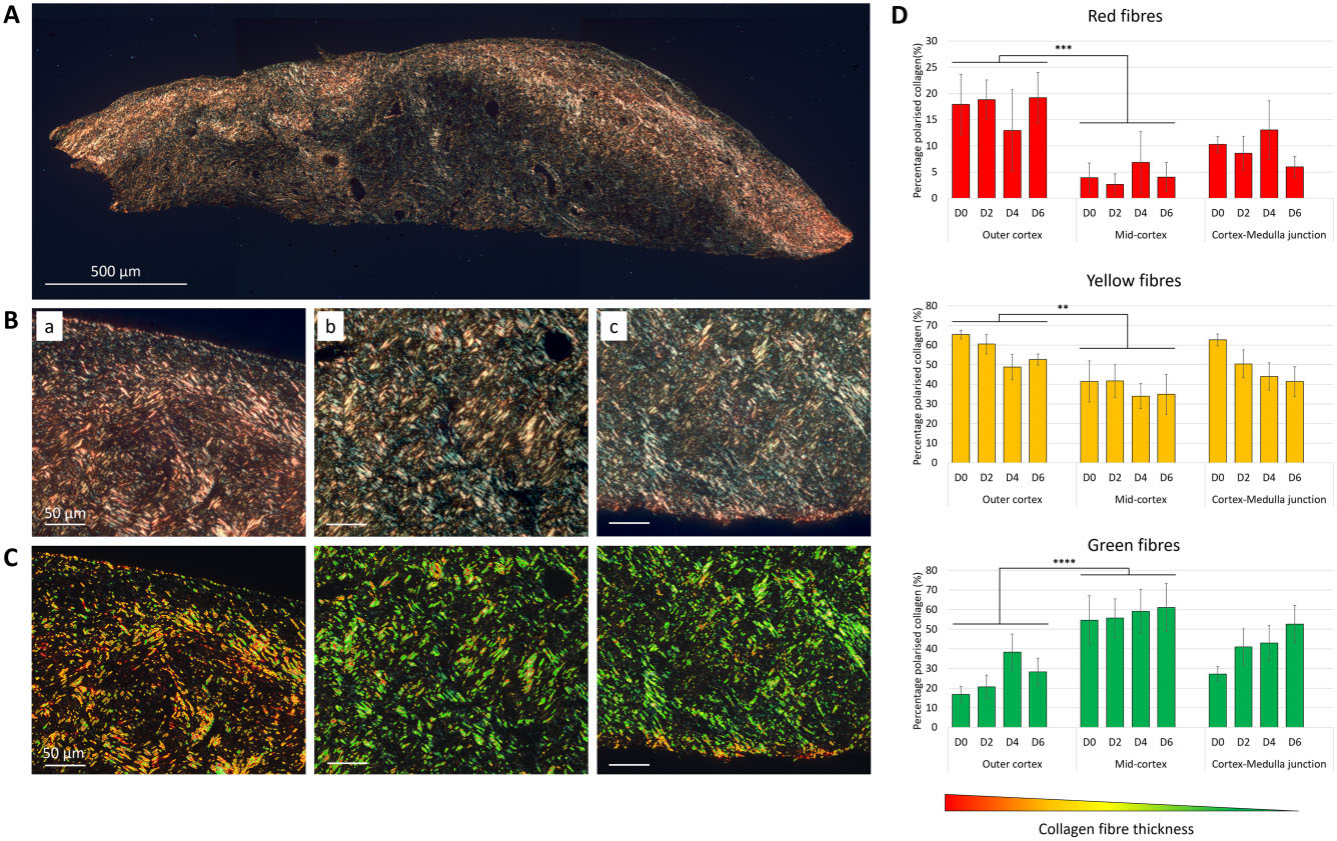
**Collagen deposition in the human ovarian cortical stroma visualized under polarized light.** (**A**, **B**) Representative PicroSirius Red (PSR) staining of an ovarian cortical section (A) and of examined cortical sub-regions (B)—outer cortex (**a**), mid-cortex (**b**), and cortex–medulla junction (**c**)—viewed under polarized light. Scale bars = 500 and 50 µm, respectively. (**C**) PSR-stained samples from (B) were analysed using a custom FIJI macro to threshold the three main colours seen under polarized light in: red, yellow, and green. (**D**) Quantification of the relative percentage of each collagen colour type (n = 6). Collagen fibre thickness varies according to its location within the cortex but is not significantly remodelled over time within each sub-region. Data are expressed as mean ± SEM, univariate general linear models. Comparison between cortical sub-regions: **P < 0.01, ***P < 0.001, ****P < 0.0001.

In addition to the biochemical properties of the ovarian cortical ECM, its micro-scale architecture, including fibres size, geometry, and organization, determines its 3D spatial configuration and thus the local biomechanical forces imposed on neighbouring cells. As collagen is the most represented matrisome-related protein in the human ovarian cortex (Ouni *et al.*, 2019), we characterized the differences in its microstructure at both the region- and culture period levels. Collagen fibres thickness and packing density were assessed under polarized light (Fig. 5A–C). Overall, the thickest (red) fibres were the least abundant in human ovarian cortex and represented ∼10% of total collagen, versus ∼48% and ∼42% for the mid-sized (yellow) and thin (green) fibres, respectively. All fibre types were significantly differentially distributed across the ovarian cortex (thick fibres—*P* = 0.002; mid-sized fibres—*P* = 0.003; thin fibres—*P* < 0.0001) but not remodelled over time (thick fibres—*P* = 0.989; mid-sized fibres—*P* = 0.075; thin fibres—*P* = 0.236) (Fig. 5D). Notably, thick and mid-size collagen fibres were predominantly found in the outer cortex and to a lesser extent in the cortex–medulla junction, while the mid-cortex retained the highest proportion of thin collagen fibres.

**Figure 6.**
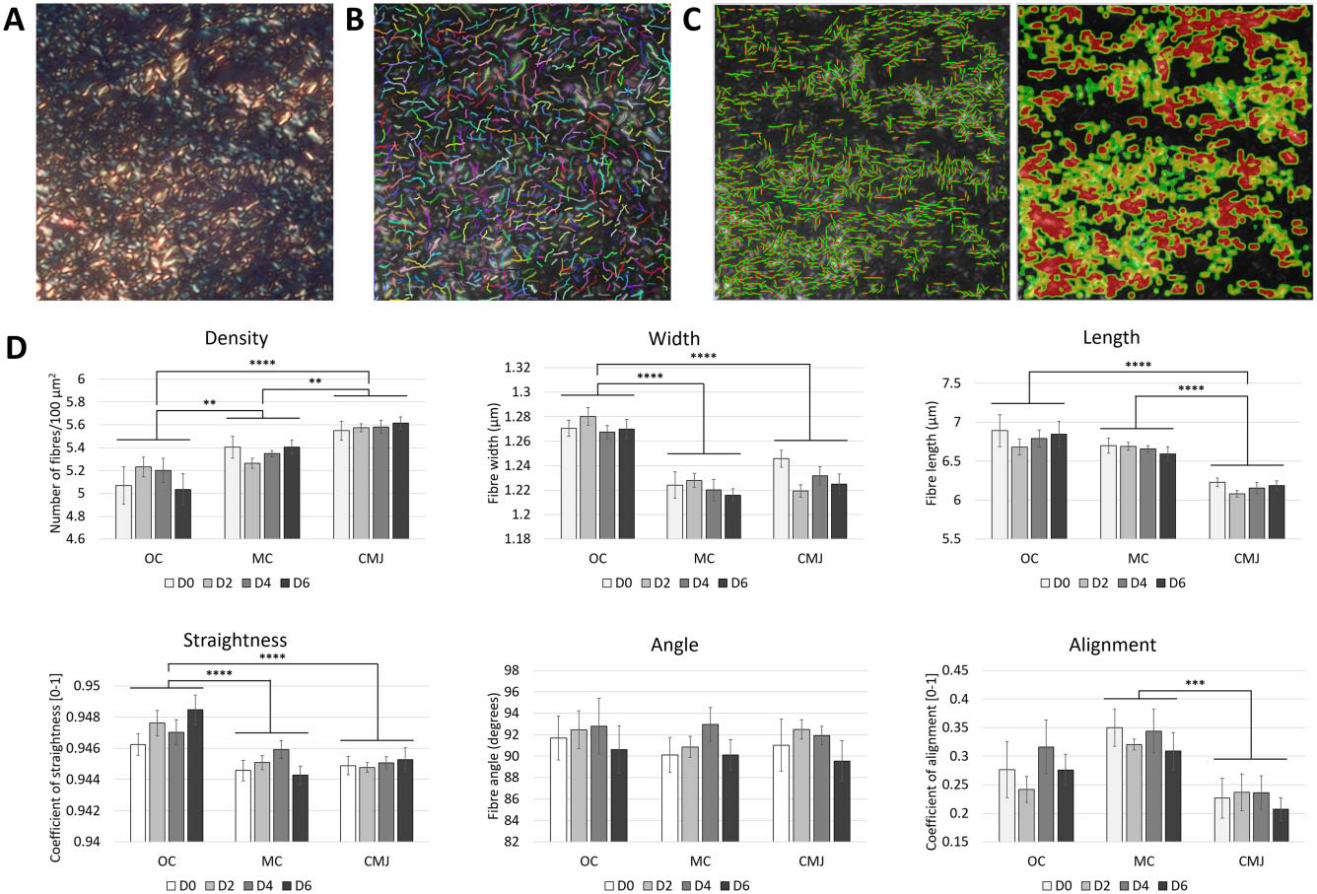
**Characteristics of the human ovarian cortical collagen architecture.** (**A**) Representative image of a region of interest stained with PicroSirius Red and viewed under polarized light. Field size = 137 × 137 µm^2^. (**B**) Graphical output from curvelet transform-fibre extraction (CT-FIRE) showing automatic extraction of collagen fibres. (**C**) Graphical outputs from CurveAlign showing fibre overlaid image that is converted into a direction heat map. (**D**) Quantification of collagen fibres density, width, length, straightness, angle, and alignment (n = 6 patients; >17 000 fibres analysed per region and day of culture). Collagen fibre metrics vary according to its location within the cortex but are not significantly remodelled over time within each sub-region. Data are expressed as mean ± SEM, univariate general linear models. Comparison between cortical sub-regions: **P < 0.01, ***P < 0.001, ****P < 0.0001.

We further quantified collagen fibre metrics, including density, width, length, straightness, angle, and alignment, in each sub-region of the ovarian cortex and throughout the culture period, using CT-FIRE and CurveAlign software (Fig. 6A–C). A total of 217 113 collagen fibres were analysed. The data revealed that the collagen fibre patterns in the human ovarian cortex have distinct signatures according to the cortical sub-region, with differences in fibre densities, widths, lengths, straightness, and alignments (Fig. 6D). Particularly, fibres from the outer cortex fibres are different to those found at the cortex–medulla junction, whilst mid-cortex fibres share features from both compartments. Overall, compared to the collagen fibres localized at the medulla border, the collagen fibres found in the outer cortex are wider (1.272 ± 0.004 versus 1.230 ± 0.004 µm; *P* < 0.0001) and longer (6.80 ± 0.08 versus 6.16 ± 0.03 µm; *P* < 0.0001), which is consistent with an increased straightness (0.9473 ± 0.0004 versus 0.9450 ± 0.0003; *P* < 0.0001) and reduced density (5.13 ± 0.07 versus 5.58 ± 0.03 fibres/100 µm^2^; *P* < 0.0001). These results mirror the increased amount of thick collagen fibres observed under polarized light, suggesting a tightly packed matrix of larger collagen fibres. In contrast, collagen fibres at the cortex–medulla junction are smaller and curvier, and more randomly organized, with less alignment compared to the mid-cortical fibres (0.23 ± 0.02 versus 0.33 ± 0.02, respectively; *P* < 0.001). The mid-cortex is composed of a mesh-like network of sparse and thin collagen fibres, in accordance with the higher proportion of green fibres under the polarized light, yet they are long and aligned in a more orderly manner than at the cortico-medullary junction. Importantly, these aspects of the collagen micro-architecture did not seem to be reshaped during the culture period, with no significant variations in the collagen morphology in each of the cortical sub-regions over time.

### Follicle spatial distribution is uneven within the cortex and shifts towards the medulla border as folliculogenesis progresses

**Figure 7.**
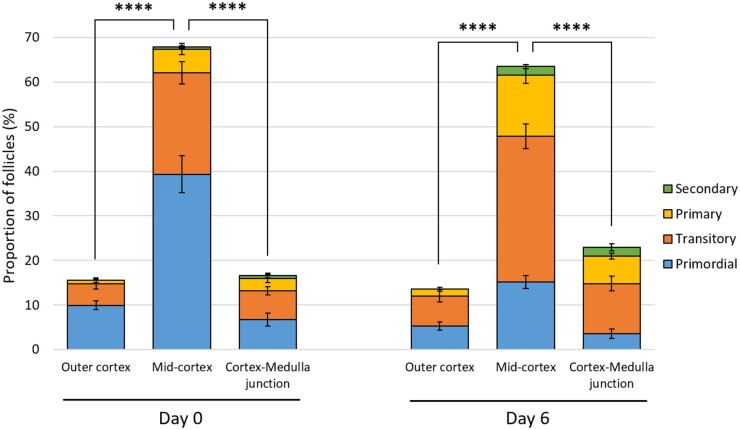
**Follicle spatial distribution within the human cortex during *in vitro* culture.** Follicles were classified according to both their developmental stage and location within the cortex at 0 and 6 days of culture (n = 6; 2805 follicles counted). Data are expressed as mean ± SEM, multivariate general linear models. ****P < 0.0001.

In order to link follicle dynamics with ovarian cortex remodelling during culture, a total of 1058 and 1747 follicles from six patients were examined at D0 and D6, respectively, and classified according to both their developmental stage and spatial position within the tissue. Follicles were not homogenously distributed throughout the ovarian cortical parenchyma, being predominantly located in the mid-cortical sub-region, representing 67.9 ± 2.9% and 63.5 ± 4.5% of the total follicle number at D0 and D6, respectively (Fig. 7). The proportion of total follicles was similar in the outer cortex and the cortex–medulla junction at D0, ranging from 15.5 ± 1.2% to 16.6 ± 2.2% total follicles, respectively (*P* = 0.949), but tended to move towards the centre of the ovary at the end of the culture period when compared to the ovarian surface, although not significant (22.9 ± 3.5% versus 13.5 ± 2.3%; *P* = 0.23).

The spatial distribution of follicles within the cortical sub-regions was also correlated with their stage of development. Although all follicular stages were predominantly located in the mid-cortex, primordial and transitory follicles were evenly distributed between the outer cortex and cortico-medullary sub-regions both at D0 (*P* = 0.706 and *P* = 0.798, respectively) and D6 (*P* = 0.643 and *P* = 0.359, respectively). In contrast, growing follicles, including primary and secondary follicles, had an initial uniform distribution between both regions (0.8 ± 0.5% versus 3.4 ± 1.1%; *P* = 0.272) but shifted towards the medulla border after 6 days of culture (1.5 ± 0.4% versus 8.1 ± 1.0%; *P* = 0.03). Moreover, no secondary follicles were ever seen in the outer cortical region.

## Discussion

It is becoming clear that primordial follicle dormancy and activation is related to the ovarian environment. Previous experiments with cultured whole mouse ovaries and large mammal cortical fragments established the significant role of the ovarian stroma in supporting primordial follicle activation and early growth (Eppig and O'Brien, 1996; Hovatta *et al.*, 1997; Wright *et al.*, 1999; O'Brien *et al.*, 2003; Telfer *et al.*, 2008; Garor *et al.*, 2009). However, how the stromal environment contributes to controlling the balance between follicle dormancy versus awakening and whether *in vitro* culture impacts this finely tuned equilibrium remains to be elucidated. Here, we showed that primordial follicle activation occurs concomitantly with a loosening of the ovarian cortex during culture, characterized by an early decrease in stromal cell density and a dynamic remodelling of the ovarian ECM both in a spatial and temporal fashion. Our data also highlight distinct collagen and elastin gradients within the ovarian cortex, likely indicating varying regional mechanical properties within the tissue. These results are corroborated with the specific signature of the collagen fibre metrics across the cortex and might be responsible for the spatio-temporal and developmental pattern of follicular distribution observed within the cortex.

*In vitro* culture of human ovarian cortical fragments containing early-stage follicles triggered a substantial activation of primordial follicle growth. This is in line with previously reported experiments performed in large mammals including humans (Wandji *et al.*, 1996, 1997; Silva *et al.*, 2004; Telfer *et al.*, 2008; Peng *et al.*, 2010; Grosbois and Demeestere, 2018). Beyond the already known signalling pathways involved in the regulation of follicle dormancy versus activation (reviewed in Grosbois *et al.* (2020)), new evidence suggests a role of the ECM in controlling follicle awakening. In this study, quantitative assessment of stromal cell density and ECM components revealed an early decrease in compactness and a dynamic ECM remodelling of cultured ovarian cortical fragments. We speculate that ovarian stretching modifies the local mechanical stress and induces a mechano-regulation feedback that leads to ECM rearrangement in order to maintain overall form and function. A similar stretch-induced ECM remodelling has been reported in cultured cells from human and mouse periodontal ligament stem cells and lung, cardiac and scleral fibroblasts (Shelton and Rada, 2007; Herum *et al.*, 2017; Pei *et al.*, 2020; Xie *et al.*, 2020), while mechanical stretching of human mammary and canine kidney epithelial cells has been linked with low cell density, modulation of Yes-associated protein/transcriptional coactivator with PDZ-binding motif activity and cell cycle entry (Aragona *et al.*, 2013; Gudipaty *et al.*, 2017). Another possibility is that *in vitro* culture indirectly triggers ECM remodelling via phosphatidylinositol 3-kinase/protein kinase B and Hippo disturbance as well as an imbalance of ECM-regulating enzymes. Indeed, both pathways are disrupted during the initial days of ovarian *in vitro* culture (Grosbois and Demeestere, 2018; Devos *et al.*, 2020) and have been shown to regulate the expression of matrix metalloproteinases (ECM-degrading proteinases) and tissue inhibitor of metalloproteinases (proteinases inhibitors) in human gastric, cartilage, and colorectal tissues and cells (Qureshi *et al.*, 2007; Yoo *et al.*, 2011; Nukuda *et al.*, 2015). As to ovarian follicles, in the ovary, fragmentation into micro-cortex triggers an imbalance in the G-actin/F-actin ratio, which disturbs the Hippo cascade of negative regulators of growth and results in follicular onset of growth (Kawamura *et al.*, 2013; Grosbois and Demeestere, 2018; Devos *et al.*, 2020). Follicles within cortical tissue prepared as solid cubes with dense stroma also show little growth initiation and slower follicle growth (Hovatta *et al.*, 1997) when compared with cortex prepared as flattened ‘sheets’ (Telfer *et al.*, 2008; McLaughlin *et al.*, 2018), supporting a connection between follicle activation, Hippo disruption and tissue loosening. It is also plausible that ECM rearrangement releases ECM-bound growth factors and increases their bioavailability, contributing to the initiation of growth.

The ovarian cortical ECM was actively remodelled during culture, as demonstrated by a gradual degradation of collagen content and a rise in elastin deposition over time, while fibronectin and laminin remained stable. Fibronectin and laminin function as bridges between ECM components to reinforce the network, and connect ECM to cells and soluble molecules within the extracellular space (Mouw *et al.*, 2014). Both proteins were detected diffusely throughout the ovarian cortex, in accordance with previous data reported in mice and humans (Berkholtz *et al.*, 2006; Heeren *et al.*, 2015; Hassanpour *et al.*, 2018), but not actively remodelled over the culture period. Furthermore, collagen confers tissues with stiffness and tensile strength, enabling resistance to deformation and rupture, while elastin confers extensibility and reversible recoil, providing the ovary with the resilience necessary to withstand the cyclic structure changes related to follicular development. Both proteins have been shown to change with age. Briley *et al.* (2016) demonstrated a positive linear correlation between age and collagen staining in mouse ovary (Briley *et al.*, 2016), also reported in pigs (Pennarossa *et al.*, 2022) and in humans whose ovarian collagen gradually accumulates from prepuberty to menopause (Ouni *et al.*, 2020), rendering the ECM stiffer and increasingly dense (Amargant *et al.*, 2020). Elastin content is lower in aged porcine ovaries compared to young ones (Pennarossa *et al.*, 2022), and in the human displays a moderate increase from the prepubertal to reproductive-age ovary, before drastically declining in menopausal women (Ouni *et al.*, 2020). Age-associated ovarian fibrosis, characterized by collagen accumulation and structure stiffening, is a contributory cause of the difficulties regarding follicular development and ovulation and has been linked with reduction of fertility (Duncan *et al.*, 2017; Amargant *et al.*, 2020; Mara *et al.*, 2020; Umehara *et al.*, 2022). Antifibrosis drugs have been shown to effectively eliminate fibrotic collagen, restore ovulation and extend female fertility in aged mice (Umehara *et al.*, 2022). The denser and more compact stromal compartment in aged ovaries, accompanied by the structure’s stiffening and loss of elasticity, could also explain the decreased rate of follicular activation from puberty to menopause (Wallace and Kelsey, 2010; McLaughlin *et al.*, 2015). Altogether, our findings suggest a turnover of the cortical ECM during culture to create a more permissive microenvironment.

The correspondence between primordial follicle activation and ovarian cortical ECM remodelling during culture may be related to the easing in the local mechanical tension. It has been shown that mouse oocytes in primordial follicles are compressed by surrounding granulosa cells secreting ECM proteins, leading to a state of high mechanical stress, and that incubation of mouse ovaries with a collagenase‐containing solution triggers activation (Nagamatsu *et al.*, 2019). ECM easing may also increase oxygen and growth factor (epidermal growth factor, fibroblast growth factor, vascular endothelial growth factor) diffusion and availability within the ovarian cortical niche (Bonnans *et al.*, 2014), indirectly promoting primordial follicle activation (Nilsson *et al.*, 2001; Danforth *et al.*, 2003; Shimamoto *et al.*, 2019; Zhang *et al.*, 2020). Furthermore, a recent study showed that granulosa cells from dormant follicles utilize a stress–response mechanism to remain arrested within a protected state, with low translation and cell cycle arrest. The authors proposed that granulosa cells respond to stress and damage by activating the integrated stress–response (ISR) and DNA damage response processes to maintain primordial follicle arrest. Resolution of this stress or damage by translational control results in a switch to an active cell cycle, leading to activation if the cell is healthy or atresia if the damage cannot be repaired (Llerena Cari *et al.*, 2021). Interestingly, the ISR activity has been shown to fluctuate among primordial follicles from the same ovary in mice, reflecting spatial differences in stress-inducing conditions (Hagen-Lillevik *et al.*, unpublished data). It is likely that the regional differences within the ovarian stroma, whether in terms of ECM composition, architecture and mechanical stress, might influence ISR action and indirectly regulate primordial follicle activation.

Our data demonstrate opposite collagen and elastin gradients across the ovarian cortex. Cortical sub-regions are also characterized by different patterns of collagen fibres in terms of thickness and packing, density, width, length, straightness and alignment. Comparable collagen fibre thickness, straightness coefficient, and angle have been previously reported in ovaries of reproductive-aged women (Ouni *et al.*, 2020, 2021). These compartmental differences within the cortex may be functional, likely conferring the tissue with distinct regional mechanical properties. Variations in ovarian ECM composition and structure have also been reported between the ovarian cortex and medulla (Laronda *et al.*, 2015; Henning *et al.*, 2019) and linked with a spatial profile of stiffness (Gargus *et al.*, 2020; Chan *et al.*, 2021; Hopkins *et al.*, 2021). Bovine ovaries assessed under atomic force microscopy displayed a rigidity gradient from the cortex (∼9 kPa) to the medulla (∼1 kPa) (Henning and Laronda, unpublished data), and evaluation of shear-wave ultrasound, which measures the speed at which shear-waves propagate through tissue, indicated a lower mean shear-wave velocity in the ovarian cortex compared to the medulla (Gargus *et al.*, 2020). A similar stiffness gradient may be expected across the distinct cortical sub-regions. Beyond its potential involvement in controlling follicle initiation of growth as discussed above, it has been suggested that ovarian follicles follow this rigidity gradient to accommodate their growth, migrating inward from the dense cortex towards the more pliant medulla and eventually back to the ovarian periphery for ovulation (Woodruff and Shea, 2011). Our spatial analysis of follicle localization shows that follicles are not uniformly distributed within the cortex, in agreement with several previous reports (Schmidt *et al.*, 2003; Schenck *et al.*, 2021), but rather are concentrated in the mid-cortical region, where the ECM is more flexible. Moreover, it confirms that follicular growth follows a geographically determined pattern, moving towards the medulla side as folliculogenesis progresses and the ovarian cortex undergoes active remodelling. This phenomenon has also been illustrated *in vivo* in mice using 3D imaging (Feng *et al.*, 2017).

This study was performed on ovarian cortical biopsies obtained from women undergoing caesarean section. Although ovulation is suppressed during pregnancy, the gonadotrophin-independent initial recruitment and early growth of follicles still occurs, as evidenced by the limited decline in serum anti-müllerian hormone (AMH) concentration with advancing gestational age (McCredie *et al.*, 2017) and our own observations confirming the presence of dormant early stage and activated, growing preantral follicles in our ovarian biopsies at the time of collection. Besides, our team has reported a wave of follicle activation following tissue stretching and *in vitro* culture in ovarian tissue from pre-pubertal girls, similar to the one observed in this study (Anderson *et al.*, 2014). This suggests that ovarian cortical ECM rearrangement is not induced by a release from pregnancy inhibition, and that similar changes might be expected in ovarian tissue from young girls and non-pregnant women.

Taken together, our data confirm that changes in the overall abundance, structure, and organization of individual ECM components directly affect the 3D spatial architecture, and potentially the biomechanical properties, of the matrix surrounding cells. It is likely that cells, including those within ovarian follicles, can sense their local environment and modulate their shape and behaviour accordingly. Future investigations should aim at clarifying the role of the ovarian ECM during folliculogenesis, and more specifically, determining which biochemical and biomechanical properties of the matrisome are necessary to regulate follicle activation. Overall, characterization of the native ovarian cortical ECM and its dynamic changes during culture will facilitate the development of more effective *in vitro* culture systems and refined bioengineered matrices for artificial ovaries, as well as contribute to improving the *in vitro* activation technique. Understanding ovarian ECM dynamics during homeostasis and pathological conditions has extensive clinical implications and becomes crucial to overcoming female infertility. Structural changes involving an aberrant ECM and a densely collagenized, thickened and stiffer cortex have been described in ovaries of patients with PCOS and ageing women (Hsueh *et al.*, 2015; Shah *et al.*, 2018; Amargant *et al.*, 2020). Moreover, patients with POI display a highly variable ovarian cortical stiffness, ranging from ∼1 to ∼11 kPa (Mendez *et al.*, 2022). This large subset of patients has in common a dysfunctional or quenched folliculogenesis, which could potentially be rescued by fine-tuning the local microenvironment. For instance, *in vitro* activation treatment is more likely to restore ovarian function in patients with POI whose ovarian cortex is stiffer (Mendez *et al.*, 2022), probably because a stiffer ovary contains more residual follicles and that fragmentation softens the local environment, releasing the follicle’s inhibition of growth. Therefore, elucidating the underlying signalling pathways and regulators that are responsive to mechanical cues becomes crucial for designing drug or cell-based therapies that aim to modulate tissue stiffness and ultimately salvage small ovarian follicles.

## Supplementary Material

dead008_Supplementary_Figure_S1

dead008_Supplementary_Figure_S2

dead008_Supplementary_Figure_S3

## Data Availability

The data underlying this article will be shared upon reasonable request to the corresponding author.
