# Supplementary material for: Spatio-temporal remodelling of the composition and architecture of the human ovarian cortical extracellular matrix during in vitro culture
Source: Hum Reprod. 2023 Jan 31;38(3):444–58. doi: 10.1093/humrep/dead008 (PMC9977129; doi:10.1093/humrep/dead008)
Supplement: dead008_Supplementary_Figure_S1 [file dead008_supplementary_figure_s1.pdf]

Human Reproduction, pp. 1–1, 2023
https://doi.org/10.1093/humrep/dead008

human reproduction

SUPPLEMENTARY DATA

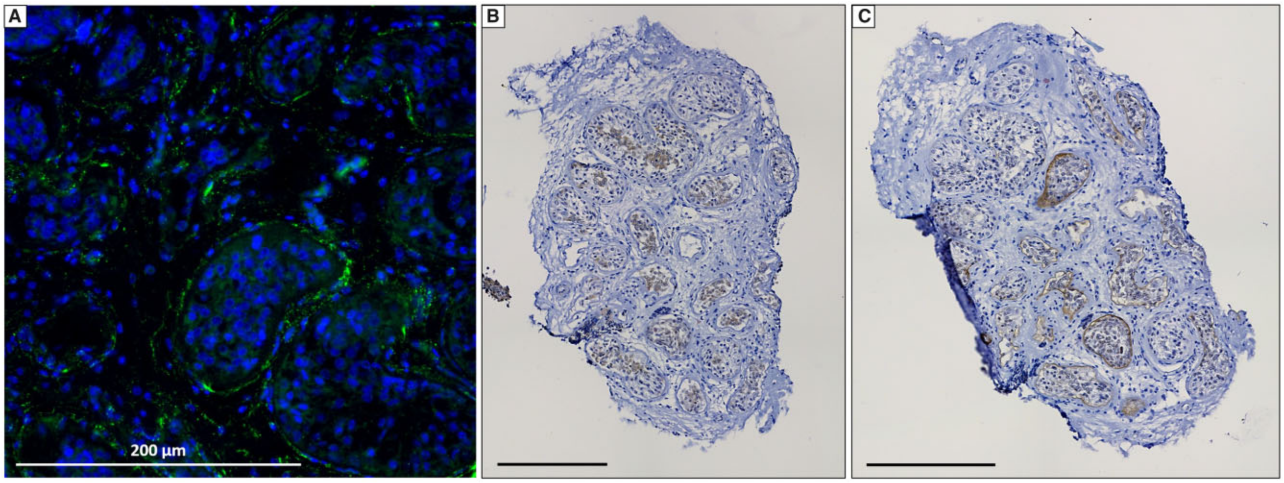

Supplementary Figure S1. Positive controls for immunostaining. Elastin (**A**), fibronectin (**B**), and laminin (**C**) are shown. Human testicular tissue was used for positive controls of all tested antibodies. Elastin stained in green, DAPI in blue, and fibronectin and laminin in brown. Scale bar = 200 µm.
