# Supplementary material for: Spatio-temporal remodelling of the composition and architecture of the human ovarian cortical extracellular matrix during in vitro culture
Source: Hum Reprod. 2023 Jan 31;38(3):444–58. doi: 10.1093/humrep/dead008 (PMC9977129; doi:10.1093/humrep/dead008)
Supplement: dead008_Supplementary_Figure_S2 [file dead008_supplementary_figure_s2.pdf]

Human Reproduction, pp. 1–1, 2023
https://doi.org/10.1093/humrep/dead008

human reproduction

SUPPLEMENTARY DATA

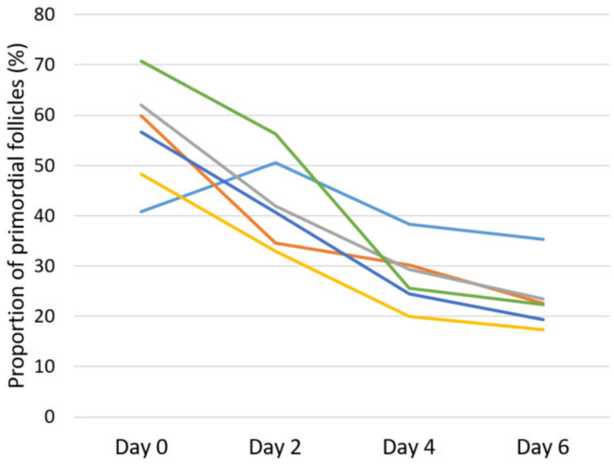

Supplementary Figure S2. Comparison of the proportion of human primordial follicles by the culture period. Spaghetti plot of the proportion of primordial follicles (%) as estimated by the day of culture. Intraclass correlation coefficient (ICC) is calculated from the random effects variance for the model. ICC is 0.144, indicating that 14.4% of the variation in the rate of primordial follicle can be explained by subject difference.
