# Supplementary material for: Spatio-temporal remodelling of the composition and architecture of the human ovarian cortical extracellular matrix during in vitro culture
Source: Hum Reprod. 2023 Jan 31;38(3):444–58. doi: 10.1093/humrep/dead008 (PMC9977129; doi:10.1093/humrep/dead008)
Supplement: dead008_Supplementary_Figure_S3 [file dead008_supplementary_figure_s3.pdf]

Human Reproduction, pp. 1–1, 2023
https://doi.org/10.1093/humrep/dead008

human reproduction

SUPPLEMENTARY DATA

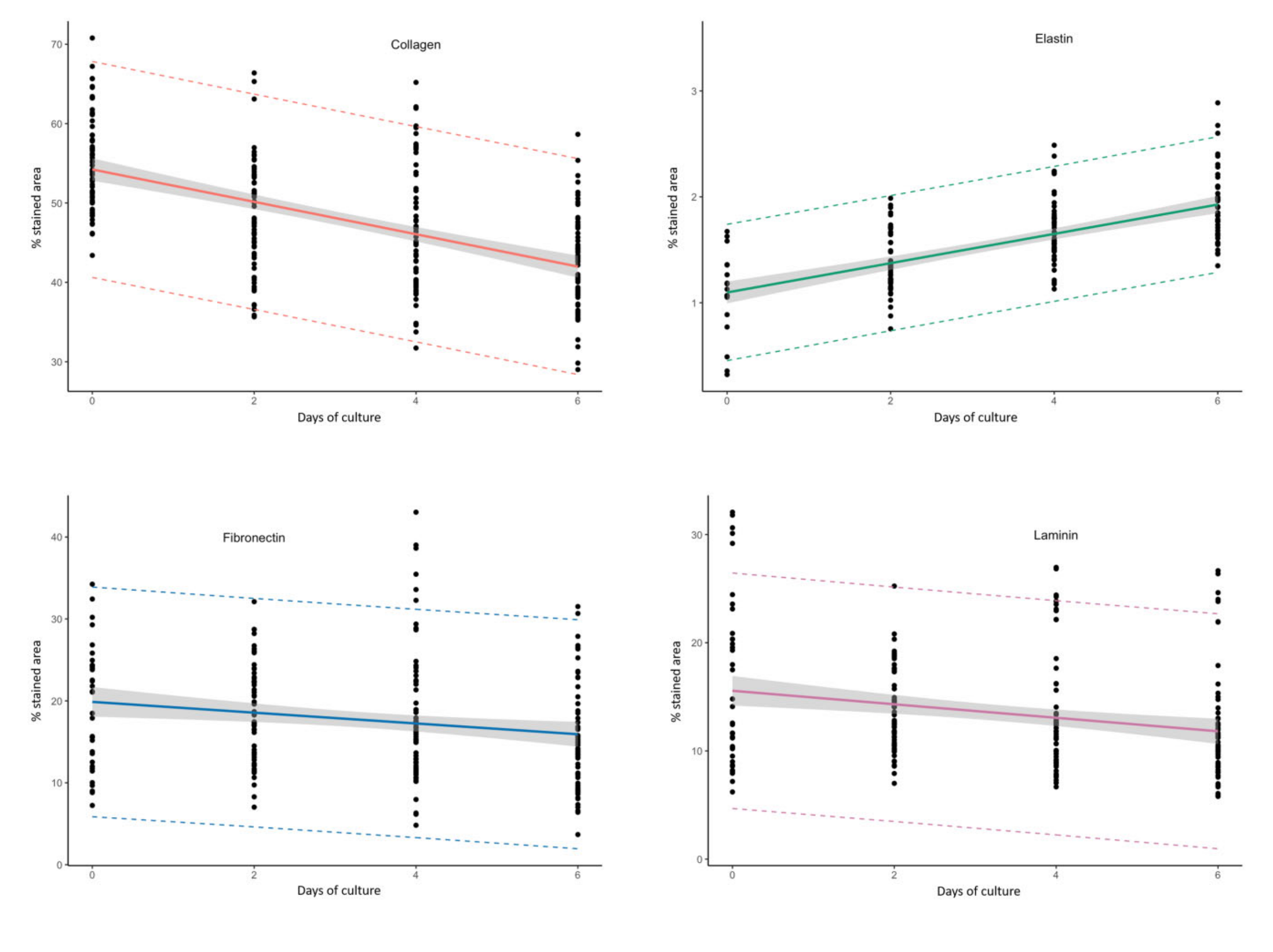

Supplementary Figure S3. Extracellular matrix components remodelling during culture. For each protein, a scatter plot with lines of best fit and 95% CI as shaded regions are depicted. Dashed lines represent the 95% prediction limits for the model, indicating that 95% of all future observations from the same underlying population are expected to fall within these limits.
